# Practice towards pesticide handling, storage and its associated factors among farmers working in irrigations in Gondar town, Ethiopia, 2019

**DOI:** 10.1186/s13104-019-4754-6

**Published:** 2019-10-30

**Authors:** Chalie Mequanint, Betelihem Getachew, Yonas Mindaye, Dagnachew Eyachew Amare, Tadesse Guadu, Henok Dagne

**Affiliations:** Department of Environmental and Occupational Health & Safety, Institute of Public Health, College of Medicine and Health Sciences, University of Gondar, Gondar, Ethiopia

**Keywords:** Associated factors, Attitude, Knowledge, Pesticide handling and storage practice, Gondar town

## Abstract

**Objectives:**

The main objective of this study was to assess pesticide handling and storage practice, and its associated factors among farmers engaged in irrigation in Gondar town, Ethiopia, 2019. Community-based cross-sectional study was used to assess pesticide handling and storage practice, and its associated factors among farmers. Simple random sampling technique was used to select study subjects. Semi-structured questionnaires were used to collect data. Data were entered using Epi Info 7.0 and exported to SPSS 20. Descriptive statistics were used for some variables. Univariate and multivariable binary logistic regression was used to test the factors associated with the outcome. Adjusted odd ratio (AOR) and 95% confidence interval (CI) with *P* value less than 0.05 were used to report association.

**Results:**

About 409 study subjects completed the semi-structured questionnaires. Among the studies subjects, 261 (63.8%) of them had poor pesticide handling and storage practice. Knowledge [AOR = 3.23 (95% CI 1.91, 5.46)], attitude [AOR = 1.77 (95% CI 1.11, 2.81) and educational status (informal education [AOR = 3.05 (95% CI 1.72, 5.42)], elementary education [AOR = 5.38 (95% CI 2.62, 11.06)], secondary education [AOR = 9.51 (95% CI 4.24, 21.32)] and certificate and above [AOR = 6.00 (95% CI 1.58, 22.78)]) were factors significantly associated with pesticide handling and storage practice. To improve the pesticide handling and storage practice, it is imperative to enhance the level of the farmer’s knowledge through training, and information dissemination in workshops.

## Introduction

Pesticides are chemicals used for deterring, destroying or controlling any pest causing harm [1, 2]. Humans come into contact with pesticides during mixing pesticides, pesticide application in the field, weeding, harvesting, and when collecting vegetables; or in their homes when pesticides are used to kill mosquitoes, cockroaches, bed bug and flies. The storage practice is believed to be poor among farmers in Ethiopia due to the knowledge gap [3, 4]. Therefore, the poor practice of storing pesticides may lead to adverse health consequences [4, 5].

A higher proportion of pesticide poisonings and deaths occur in developing countries where there are inadequate occupational safety standards, lack of use of personal protective equipment (PPE), inadequate hygienic facilities, illiteracy, and insufficient knowledge of pesticide hazards [6–9]. Furthermore, without sufficient ventilation and adequate PPEs, pesticides fumes may be inhaled and dermally absorbed into the human body [10].

Improper use of pesticides affect the natural environment such as water, soil and air and from pesticide leaching, runoff, and spray drift wildlife, fish, plants, and other non-target organisms may be affected [11]. Environmental contamination can lead to human exposure through consumption of residues of pesticides in food and drinking water [11, 12].

Pesticide exposure can cause health problems such as human carcinogens, dermatitis, kidney disorders, endocrine disruption, reproductive effects, blurred vision, neuropsychiatric disturbance and memory loss [6, 13–15]. In particular, pregnant women and children are vulnerable groups. Pesticides pose a particular risk to children as their internal organs are still developing, still maturing enzymatic, metabolic, and immune systems [14].

Developed countries have systems already in place to register pesticides and regulating their trade and use. However, in developing countries, such kinds of systems are weaker [16]. Developing countries do not have effective monitoring systems in place to assess the extent of pesticide poisonings and the majority of cases are unreported [16]. Studies showed that most pesticides handlers have no sufficient knowledge regarding the safe use and handling of the pesticides [3, 9, 10, 17, 18].

To our capacity, there have been scarce studies conducted in Ethiopia regarding pesticide handling and storage practice [3, 8]. However, they either emphasized on knowledge and attitude only or on some specific cases of pesticide practice. Moreover, irrigation farming is steadily increasing in Ethiopia including in Gondar which may contribute to an increased application of pesticide.

Therefore, the main objective of the study was to assess pesticide storage and handling practice and its associated factors among farmers working in irrigation in Gondar town, Ethiopia, 2019.

## Main text

### Methods

#### Study design, area and period

This community-based cross-sectional study was conducted from April to May 2019 among farmers working in irrigation in Gondar town, Ethiopia. The total number of population of Gondar was estimated to be 324,000 and among these 480 were farmers engaged in irrigation.

#### Study population

All irrigation farmers in the selected area who were present during data collection and voluntary were included.

#### Operational definitions

*Pesticide storage and handling practices* The respondents who scored less than or equal to the mean value of the responses for 22 pesticide handling storage practice-related questions were considered having “poor level of practice”. Those who scored more than the mean value were considered as having a “good level of practice.”

*Pesticide handling, and storage knowledge level* The respondent who scored less than or equal to the mean value of the responses for 10 pesticide handling, and storage knowledge-related questions were considered having “poor level of knowledge.” Those who scored more than the mean value were considered as having a “good level of knowledge.”

*Pesticide handling and storage attitude* the respondent who scored less than or equal to the mean value of the response for 5 pesticide handling, and storage attitude related questions were considered having “poor level of attitude.” Those who scored more than the mean value were considered as having a “good level of attitude.”

#### Sample size determination and procedure

The sample size was determined using a single population proportion formula. The minimum sample size (n) for the study was calculated as follows. Where the critical value is ($Z_{a/2}$) is 1.96 at 95% confidence interval, d (degree of precision) was 0.05 and P (proportion of good pesticide handling practice based on previous study 55.9%) [4].

$$n = \frac{{\left( {z_{{{\raise0.7ex\hbox{$\alpha $} \!\mathord{\left/ {\vphantom {\alpha 2}}\right.\kern-0pt} \!\lower0.7ex\hbox{$2$}}}} } \right)^{2} p(1 - p)}}{{d^{2} }} = \frac{{\left( {1.96} \right)^{2} 0.559\;(1 - 0.559)}}{{0.05^{2} }} = 379.$$

Adding 10% for any unexpected events 10% of 379 equals to 37.9 ≈ 38 so, the total Samples were 379 + 38 = 417.

From the total 480 farmers engaged in irrigation, 417 were selected by simple random sampling technique from three river basins namely Qeha, Shinta and Angereb. One hundred and fifty-seven out of the 180 farmers in Angereb, 131 out of 151 farmers in Shinta, and 129 out of the 147 farmers in Qeha were proportionally chosen.

#### Data collection tool, method, quality control, and processing and analysis

The data were collected by interviewing using questionnaires which comprised questions about pesticide storage and handling practice, sociodemographic characteristics, knowledge and attitude. The quality of data was assured by proper designing of the questionnaire. The questionnaires were prepared in English, translated to Amharic and translated back to English to check for consistency. The data were checked for completeness and entered using Epi info version 7.0 and exported to SPSS version 20. Univariate and multivariable logistic regression was computed to test the factors associated with pesticide handling and storage practice.

### Results

#### Sociodemographic characteristics

Four hundred and nine farmers participated in the study. The majority of the respondents (90.7%) were male and the mean (± SD) age of study participants was 35.99 ± 9.5 years (Table 1).

**Table 1:** Sociodemographic characteristics of farmers engaged in irrigation, Gondar town, 2019 (n = 409)

Variable	Frequency (n)	Percent (%)
Sex		
Male	371	90.7
Female	38	9.3
Religion		
Orthodox	355	86.8
Muslim	45	11.0
Protestant	7	1.7
Others	2	0.5
Marital status		
Unmarried	102	24.9
Married	307	75.1
Age		
16–29	120	29.3
30–35	76	18.6
36–40	97	23.7
≥ 40	116	28.4
Level of education		
Cannot read and write	159	38.9
Read and write only	139	34.0
Primary	55	13.4
Secondary	45	11.0
Certificate and above	11	2.7
Income		
300–2000	121	29.6
2001–3000	110	26.9
3001–4000	96	23.5
4001–10,000	82	20.0
Family size		
1–4	172	42.1
> 4	237	57.9

#### Pesticide handling and storage practice

About 148 (36.2%) subjects had self-reported good pesticide handling and storage practice. Similarly, 161 (39.4%) and 201 (49.1%) of farmers reported a good level of knowledge and attitude regarding pesticide handling and storage, respectively (Additional file 1: Fig. S1).

Sixty-seven (16.4%) of participants reported that they used empty pesticide containers for storing food and non-food materials. About 322 (78.7%) reported that they were exposed for pesticide during the application, and 95 (23.2%) of the respondents reported that they mix pesticide in front of other people including children (Table 2). Responses for specific pesticide storage and handling knowledge and attitude questions are indicated on tables (Additional file 2: Table S1 and Additional file 3: Table S2) respectively.

**Table 2:** Pesticide handling and storage practice of farmers in Gondar town, 2019 (n = 409)

Variables	Frequency (n)	Percentage (%)
Overall practice		
Poor	279	63.8
Good	130	36.2
Use of empty pesticide containers	67	16.4
Purpose of use of empty pesticide containers (n = 67)		
Containing non-food items	22	5.4
Containing food items	45	11.0
Exposed for pesticide during application	322	78.7
Type of PPE used during handling		
Normal cloth	360	88.0
Gloves	31	7.6
Rubber boot	67	16.4
Hat	79	19.3
Facemask	31	7.6
Respirator	18	4.4
Full body cover	40	9.8
Mix pesticides inside the house	105	25.7
Mix pesticide in front of other people including children	95	23.2
Where do you dispose empty pesticide container?		
On farm area	206	49.6
Place in trash or dumpster	188	46
Bury on farm	55	13.4
Where do you store left over pesticide after application?		
Generally in the house	157	38.4
Farming area	165	40.3
Kitchen	27	6.6
Bed room	55	13.4
Others^a^	5	1.2

^a^Anywhere out of house and farm area

#### Factors associated with pesticide storage and handling practice

Marital status, income, education, knowledge and attitude of farmers about pesticide handling and storage were factors with *P* value < 0.2 in the univariate analysis which are candidates for the multivariable logistic regression. In the multivariable regression analysis only educational status, knowledge and attitude were significantly associated. The study subjects with informal education were 3.05 times more likely to have better pesticide handling and storage practice [AOR = 3.05, 95% CI (1.72, 5.42)] as compared with those who cannot read and write. Whereas respondents who had elementary education were 5.38 times more likely to report better pesticide handling and storage practice [AOR = 5.38, 95% CI (2.62, 11.06)] compared to those who cannot read and write. The study subjects with a certificate and above educational status were 6 times more likely to have better pesticide handling and storage practice [AOR = 6, 95% CI (1.58, 22.78) as compared with those who cannot read and write. The study participants with good knowledge had 3.23 times more likelihood of better practice [AOR = 3.23, CI 95% (1.91, 5.46)] about pesticide handling and storage as compared with those having poor knowledge. Farmers who have desirable/good attitude reported 1.77 times more likely better practice [AOR 1.77, 95% CI (1.11, 2.81)] as compared those with undesirable/poor attitude (Table 3).

**Table 3:** Univariate and multivariable logistic regression analysis of factors associated with pesticide handling and storage practice among farmers engaged in irrigation, Gondar town, 2019

Variables	Categories	Practice		COR (95% CI)	AOR (95% CI)
		Good (%)	Poor (%)		
Marital status	Unmarried	44 (43.1)	58 (56.9)	1	1
	Married	104 (33.9)	203 (66.1)	1.48 (0.43, 1.07)	–
Education	Cannot read and write	24 (15.1)	135 (84.9)	1	1
	Informal education	53 (38.1)	86 (61.9)	3.47 (1.99, 6.03)	3.05 (1.72, 5.42)***
	Elementary	32 (58.2)	23 (41.8)	7.83 (3.93, 15.60)	5.38 (2.62, 11.06)***
	Secondary	32 (71.1)	13 (28.9)	13.85 (6.36, 30.12)	9.51 (4.24, 21.32)***
	Certificate and above	7 (63.6)	4 (36.4)	9.84 (2.68, 36.23)	6.00 (1.58, 22.78)***
Income	300–2000	39 (32.2)	82 (67.8)	1	1
	2001–3000	38 (34.5)	72 (65.5)	1.11 (0.64, 1.92)	–
	3001–4000	34 (35.4)	62 (64.6)	1.15 (0.66, 2.03)	–
	4001–10,000	37 (45.1)	45 (54.9)	1.73 (0.97, 3.08)	–
Knowledge	Good	26 (16.1)	135 (83.9)	5.03 (3.09, 8.19)	3.23 (1.91, 5.46)**
	Poor	122 (49.2)	126 (50.8)	1	1
Attitude	Good	90 (44.8)	111 (55.2)	2.10 (1.39, 3.16)	1.77 (1.11, 2.81)*
	Poor	58 (27.9)	150 (72.1)	1	1

* P < 0.05, ** P < 0.01, *** P < 0.001 Hosmer–Lemeshow-good-of fit 0.906

### Discussion

The knowledge, attitudes and practice regarding pesticide usage and related health problems among farmers engaged in irrigation have been left neglected in developing countries [4].

In this study, only 36.2% with 95% CI (31.5%, 40.8%) of the respondents had good pesticide handling and storage practice. The overall good practice in the current study was higher than previous studies conducted in Kenya (15%) [9] and in Tanzania (21%) [19]. This discrepancy could be due to the difference in socio-demographic factors, study setting and educational level of study subjects.

Poor pesticide handling practice may expose the farmers to a number of pesticide-related health symptoms [20, 21].

In this study, the level of good practice of disposing of empty pesticide containers was in line with a study conducted at Rift Valley in Ethiopia (97%) [3]. On the other hand, the good practice of empty pesticide container handling in the current study is lower than that of a study in Zimbabwe (20.2%) [22], in El Salvador (63%) [6] and in Nigeria (55.8%) [23]. This problem could be associated either with their limited level of knowledge about pesticide containers that residual chemicals still present in the container or lack of hazardous collection site easily accessible by the farmers. Discarding pesticide containers arbitrarily after emptying may risk ground or surface water due to the leakage of residual pesticides [24]. A previous study has shown that an empty container contains 2% pesticide left in the packaging material [3]. Hence, it is important to raise the level of awareness of the farmers about the importance of dumping the empty containers in a specified safe place. Furthermore, it is essential that the city authorities to furnish a centralized hazardous waste collection site for the farmers and need to have a continuous monitoring system about the collection practice.

Among the participants, 38.4% stored the purchased pesticide with the bulk of other household materials at their home. The absence of separate storage site may lead to a risk of contamination and thereby health risks. After the pesticide application, 16.4% the subjects said that they reuse the empty containers. Studies conducted in Nigeria found out that 74% and in Tanzania 68% of the farmers have suffered at least one health symptom due to the poor pesticide handling practice [5, 7, 18]. Improper disposal of pesticide containers is a totally unsafe practice [25, 26].

A significant number of farmers (23.2%) responded that they practice mixing pesticide in front of people including children. This may lead to potential health risks for the mixer and people in a close range. In particular, children are more vulnerable as they are sensitive towards low dose of pesticide contamination [27, 28]. Their skin surface area is bigger and hence a potential dermal absorption, and also their brain, metabolizing enzymes and immune system are still growing or sensitive [14]. A systematic review showed a strong correlation between pesticide exposure and Parkinson’s disease, Alzheimer’s disease and other neurodevelopmental disorders among children [29]. There is also evidence which links pesticide exposure to pediatric leukaemia [30].

Furthermore, 90.2% of respondents have revealed that they did not use personal protective equipment (PPE) during practising pesticide mixing and application. This result is better than a previous study [6] finding that reported all the study subjects did not use PPE. However, the current result is lower than compared to reports from Kuwait (58%) [18], Ghana (30.9%) [31], and Nepal (84%) [32]. Another study has shown that farmers who use PPE during mixing and spraying pesticide were 82% less likely to report acute poisoning [10].

Cost of PPE has been reported as a significant factor behind farmers’ pesticide usage without PPE [7, 31]. In general, the absence of PPE may lead to a potential exposure through dermal absorption and inhalation thereby increasing the risk of a long term health effect [15].

The educational status, knowledge and attitude of respondents were predictors of their pesticide handling and storage practice. Similarly in another study pesticide handlers with knowledge about pesticide health risks had better practice during pesticide handling [33]. A previous study revealed that farmers with a higher level of education were less likely to store pesticide at home [19]. In general, the knowledge gap due to lack of information or training can pose serious exposure and health risk for the farmers [9]. To improve the pesticide handling and storage practice, it is imperative to enhance the level of the farmer’s knowledge through training, and information dissemination in workshops.

### Conclusions

The pesticide storage and handling practice of farmers engaged in irrigation in Gondar town were poor. Educational status, knowledge, and attitude were identified as the factors having a strong association with pesticide handling and storage practice among farmers working in irrigation.

## Limitation of the study

This study might be affected by social desirability bias even though techniques to reduce bias has been employed. The inherent weakness of a cross-sectional study that fails to establish cause and effect relationship results difficulty to identify the true determinants of practice.

## Supplementary information

**Additional file 1: Table S1.** Knowledge of farmers about pesticide storage and handling practice in North West Ethiopia in Gondar town April 2019 (n = 409).

**Additional file 2: Table S2.** Attitude of farmers pesticide storage and handling practice in North West Ethiopia in Gondar town April 2019 (n = 409).

**Additional file 3: Fig. S1.** Knowledge, attitude, and practice level of the study participants regarding pesticide handling and storage among farmers in Gondar, Ethiopia, 2019 (n = 409).

## Data Availability

The dataset in the current study is available from the corresponding author upon reasonable request.

## Abbreviations

AOR: adjusted odds ratio
COR: crude odds ratio
CI: confidence interval
EPI Info: epidemiological information
PPE: personal protective equipment
SPSS: statistical package for social sciences
