# Supplementary material for: Practice towards pesticide handling, storage and its associated factors among farmers working in irrigations in Gondar town, Ethiopia, 2019
Source: BMC Res Notes. 2019 Oct 30;12:709. doi: 10.1186/s13104-019-4754-6 (PMC6820978; doi:10.1186/s13104-019-4754-6)
Supplement: Supplementary file 2 — Additional file 2: Table S2. Attitude of farmers pesticide storage and handling practice in North West Ethiopia in Gondar town April 2019 (n = 409). [file 13104_2019_4754_MOESM2_ESM.docx]

| Variables/Questions | | Frequency (n) | Percentage (%) |
| --- | --- | --- | --- |
| Do you know the health impacts of pesticide use? | Yes | 392 | 95.8 |
|  | No | 17 | 4.2 |
| Do you read and understand instructions on the pesticide containers? | No | 202 | 49.4 |
|  | Yes | 207 | 50.6 |
| Is there a health problem associated with pesticide handling? | No | 178 | 43.5 |
|  | Yes | 231 | 56.5 |
| Do you know pesticide affects the environment? | No | 19 | 4.6 |
|  | Yes | 390 | 95.4 |
| Do you know the problems of expired pesticide? | Yes | 223 | 54.5 |
|  | No | 186 | 45.5 |
| Is weathering condition affect pesticide during application? | No | 72 | 17.6 |
|  | Yes | 337 | 82.4 |
| Is sun light affect pesticide during application spraying? | No | 155 | 37.9 |
|  | Yes | 254 | 62.1 |
| Do you know the problems of expired pesticide? | Yes | 223 | 54.5 |
|  | No | 186 | 45.5 |
| Do pesticides affect non target living things? | Yes | 339 | 82.9 |
|  | No | 70 | 17.1 |
| Do you know that you have to read safety instruction while using and storing pesticides? | Yes | 226 | 55.3 |
|  | No | 183 | 44.7 |
