# Supplementary material for: Practice towards pesticide handling, storage and its associated factors among farmers working in irrigations in Gondar town, Ethiopia, 2019
Source: BMC Res Notes. 2019 Oct 30;12:709. doi: 10.1186/s13104-019-4754-6 (PMC6820978; doi:10.1186/s13104-019-4754-6)
Supplement: Supplementary file 3 — Additional file 3: Fig. S1. Knowledge, attitude, and practice level of the study participants regarding pesticide handling and storage among farmers in Gondar, Ethiopia, 2019 (n = 409). [file 13104_2019_4754_MOESM3_ESM.docx]

| Variables/ Questions | | Frequency(n) | Percent (%) |
| --- | --- | --- | --- |
| Do you agree that pesticide storage should be far from kitchen area? | Strongly agree | 107 | 26.2 |
|  | Agree | 257 | 62.8 |
|  | Disagree | 33 | 8.1 |
|  | Strongly disagree | 12 | 2.9 |
| Do you agree that indiscriminate disposal of empty pesticide containers result in health problem? | Strongly agree | 86 | 21.0 |
|  | Agree | 215 | 52.6 |
|  | Disagree | 100 | 24.4 |
|  | Strongly disagree | 8 | 2.0 |
| Do you agree that pesticide storage in house needs serious concern and greater care? | Strongly agree | 82 | 20.0 |
|  | Agree | 292 | 71.4 |
|  | Disagree | 32 | 7.8 |
|  | Strongly disagree | 3 | 0.7 |
| Do you agree that good pesticide storage helps to reduce health problem associated with pesticide exposure? | Strongly agree | 96 | 23.5 |
|  | Agree | 287 | 70.2 |
|  | Disagree | 23 | 5.6 |
|  | Strongly disagree | 3 | .7 |
| Do you think that exposure to pesticide does not cause health problem? | Strongly agree | 22 | 5.4 |
|  | Agree | 84 | 20.5 |
|  | Disagree | 218 | 53.3 |
|  | Strongly disagree | 85 | 20.8 |
