# Supplementary material for: Practice towards pesticide handling, storage and its associated factors among farmers working in irrigations in Gondar town, Ethiopia, 2019
Source: BMC Res Notes. 2019 Oct 30;12:709. doi: 10.1186/s13104-019-4754-6 (PMC6820978; doi:10.1186/s13104-019-4754-6)
Supplement: Supplementary file 3 [file 13104_2019_4754_MOESM1_ESM.docx]

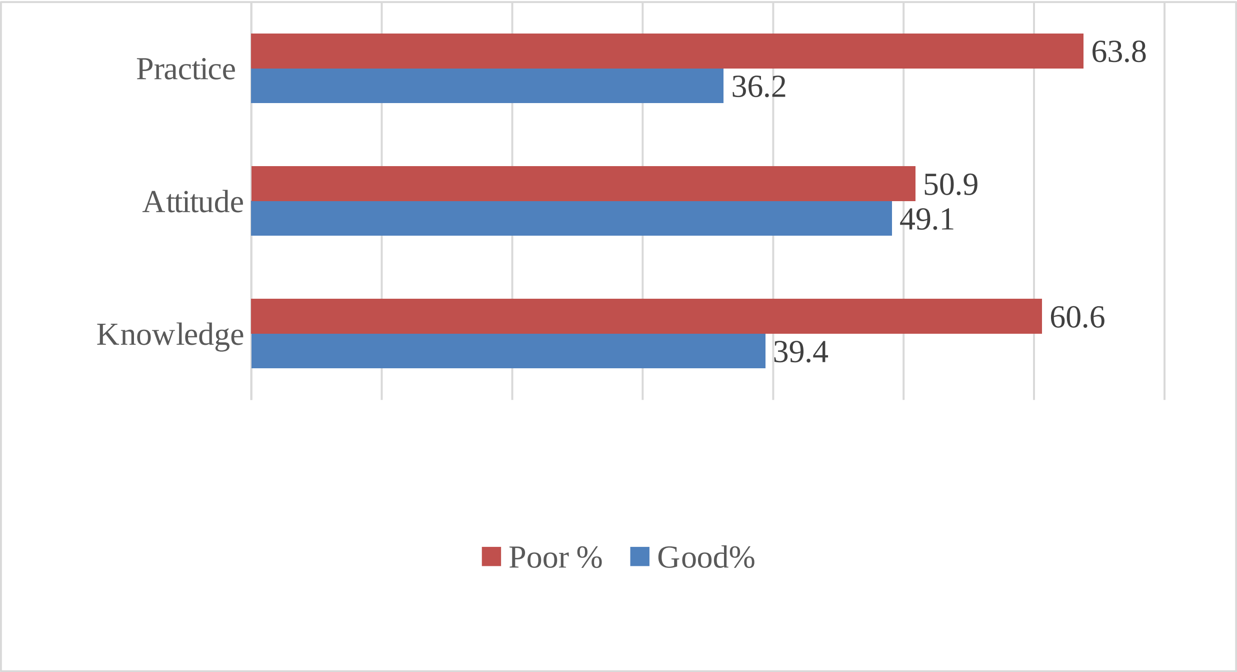
Practice
63.8
36.2
Attitude
50.9
49.1
Knowledge
60.6
39.4
Poor %
Good%
